# The removal of black ink via *Emericella quadrilineata* as a green alternative technique to recycling ink waste papers

**DOI:** 10.1371/journal.pone.0324022

**Published:** 2025-05-29

**Authors:** Mari Sumayli, Abdullah Mashraqi, A. El-Shabasy, Ugur Azizoglu, Sally A. Ali

**Affiliations:** 1 Department of Biology, College of Science, Jazan University, Jazan, Kingdom of Saudi Arabia; 2 Department of Crop and Animal Production, Safiye Cikrikcioglu Vocational College, Kayseri University, Kayseri, Türkiye; 3 Genome and Stem Cell Research Center, Erciyes University, Kayseri, Türkiye; 4 Department of Botany and Microbiology, Faculty of Science, Helwan University, Cairo, Egypt.; Washington State University, UNITED STATES OF AMERICA

## Abstract

In order to fight deforestation, biological methods of recycling printed waste papers must be used. In addition to identifying and isolating *A*. *quadrilineatus*, the current study attempts to ascertain the best physiological conditions and mechanisms underlying this species’ ability to deink. Five isolates such as *Cladosporium sp*., *Aspergillus sp*., *Fusarium sp*., *Penicillium sp*., and *Rhizopus sp*. isolated from soil containing ink remains using Bushnell and Hass media carried out the deinking tests. SEM, FT-IR, the molecular method, and factors affecting ink eradication were all carried out. The deinking of ink-loaded filter paper, Langmuir and Freundlich adsorption isotherms, and *A*. *quadrilineatus* enzyme activities were also investigated in this work. Ninety per percentage of the black ink was removed by *A*. *quadrilineatus*. Six days later, under ideal conditions (pH 6, temperature 30°C, initial ink concentrations of 20,000 mg L ⁻ ¹, and inoculum dose of three fungal discs), the optimal deinking percentage from solution through a culture of *A*. *quadrilineatus* reached up to 97%. The deinking mechanism of *A*. *quadrilineatus* was shown by SEM and FT-IR studies. A good level of agreement between the Langmuir adsorption isotherm model and the adsorption process was shown by the Freundlich and Langmuir adsorption isotherms. Otherwise, on agar plates, *A*. *quadrilineatus* demonstrated its capacity to manufacture the enzymes lipase and xylanase. Overall, the results indicated that *A*. *quadrilineatus* may open up new possibilities for recycling printed waste papers.

## Introduction

Forests and the ecosystem are being destroyed because of the rising need for paper [1]. Researchers have made effective attempts to recycle wastepaper to combat the continuously rising demand and environmental degradation [2].

Many of the traditional deinking procedures use huge amounts of chemicals, which results in the production of highly contaminated wastewater [3,4]. Processes such as pulping, flotation, cleaning, and bleaching used in paper recycling [5]. The main ingredients of printing ink include dyes, pigments, resins, binders, solvents, and optional additions [6]. Toner a powder mixture is a common component of toner cartridges for laser printers and photocopiers. Originally consisting primarily of granulated plastic, the mixtures simply included carbon powder and iron oxide. Today, some mixtures include polypropylene, fumed silica, and other minerals for triboelectrification [7]. As a substitute for plastic derived from petroleum, toner can also be produced from plastic derived from plants [8]. Because of the heat from the fuser, the toner particles melt and stick to the paper.

Deinking, the process of printing ink removal from paper, is necessary to obtain pulp. Deink typically accomplished by chemical bleaching although the emission of the chemicals during the process frequently causes environmental damage. There have been numerous attempts to provide environmentally friendly methods for deinking wastepaper [9]. Comparing biological therapy to other traditional techniques, such as physical and chemical ones, typically reveals it to be the most environmentally friendly and cost-effective option.

Many studies have observed that microorganisms like bacteria, yeasts, algae, and fungi may remove printing inks and colors [10]. In addition to degrading organic contaminants, microorganisms can also biosorb them.

Until balance is achieved, biosorption keeps going. Fungal biosorbents have the benefit of having a high percentage of cell wall material, which gives them the great biosorption capacity and a range of functional groups [11]. The main factors influencing ink removal difficulty are ink type, printing technique, and fibre type. Newspapers printed are one example of a paper type that can be deinked using conventional methods. A significant technological barrier to using recycled paper more frequently is still ink removal. To comply with environmental requirements, many of the traditional deinking techniques use significant amounts of chemicals, which raise the expense of wastewater treatment. Large quantities of liquid and solid waste produced during the deinking processes.

Deinking facilities would benefit from more efficient and less polluting disposal techniques. Then enzyme-assisted deinking techniques could be an environmentally beneficial substitute for current alkaline deinking procedures [12–14]. One of the biological methods of the deink using the cellulase enzyme which used in the replacement of conventional chemical deinking methods [15].

In the deinking of paper pulp, the most powerful microbial enzymes are cellulase and xylanase. Due to its affordability and environmental friendliness, enzymatic deinking is currently popular and has drawn interest from numerous industries [16,17]. It also produces fewer toxic effluents making the removal of toxic content from the effluent easier [18].

For the enzymatic deinking of different waste papers, a number of enzymes created by different bacteria and fungi, including cellulase, hemicellulase, laccase, pectinase, esterase, α-amylase, and ligninolytic enzymes, have been utilized either alone or in combination [19]. This study’s goal is to remove the toner powder ink from ink-loaded cellulose paper as an attempt to recycle waste paper after optimizing its removal from liquid solution using *Aspergillus quadrilineatus* and investigating the removal mechanism, which will allow for more affordable, environmentally acceptable, and widely accepted eco-friendly methods.

## Materials and methods

### Chemicals

**Fig 1:**
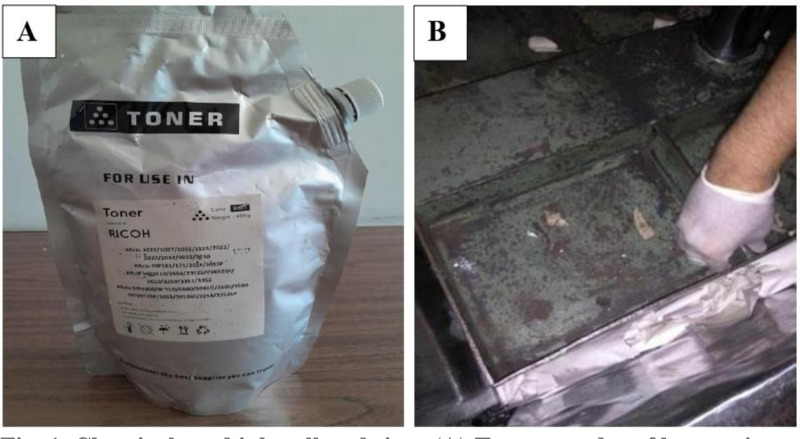
Chemicals and ink polluted sites. **(A)** Toner powder of laser printers. **(B)** Ink polluted sites at the Libraries area of Ben al-Sarayat, Cairo, Egypt.

Black toner powder ink, as shown in (Fig 1A), was purchased from Ink Outlet Cairo, Egypt. Potato dextrose agar was used to keep *A*. *quadrilineatus* a live [20]. Bushnell and Hass media was provided by a mycology lab at the “Department of Botany and Microbiology, Faculty of Science, Helwan University.” It contained (g/l) MgSO₄ (0.2), CaCl₂ (0.02), KH₂PO₄ (1), NH₄NO₃ (1), FeCl₃ (0.05), glucose (0.01), yeast extract (0.005), agar (15), and pH 7 [21].

## Methods

### Fungal isolation

As shown in (Fig 1B), fungi were isolated from ink-polluted soil that was collected from the surface soil of ink-polluted sites at the Libraries area of Ben al-Sarayat in Cairo, Egypt (no permits were required because the Libraries area of Ben al-Sarayat does not necessitate any permits, and it provided us with soil contaminated by ink). Serial dilution method was used for the isolation of fungi [22]. In a nutshell, 10 g of soil contaminated with ink residues was sieved through mesh with a 2 mm pore size sieve, suspended in a flask containing 90 mL of sterile water, and incubated at 28°C on an orbital shaker (150 rpm) for 30 min. After that, one mL of the suspension was transferred to a brand-new tube and gradually diluted to 10^-5^. To isolate fungi, 20000 mg L ⁻ ¹ of black toner powder ink was employed in the Bushnell and Hass medium and incubated at 28°C for 5–7 days. Five distinct fungi isolates, known as isolated colonies, were isolated and purified through inoculation on potato dextrose agar plates, then incubation for 5 days at 28°C.

### Screening and identification methods

The five fungal isolates’ deinking abilities tested using the procedure [6]. Erlenmeyer conical flasks containing fifty mL of Bushnell and Hass’s broth and 20000 mg L ⁻ ¹ of black toner powder ink were each inoculated with a one mL aliquot of spore suspension (10^5^ spores/ mL) suspension. Adjust the pH to 7 before autoclaving. The culture kept in an incubator for seven days at a temperature of 28°C. After 7 days, the solution withdrawn to analyze the UV-Vis spectra and quantify the deinking ability (percentage) using equation (1). As reported by Chen et al. [11] the morphological characteristics of the isolated fungi were examined using a 5-day-old fungal culture. Then a lactophenol cotton blue wet mount slide was created [23]. The morphological features (color, texture, and appearance) and microscopic features using a light microscope (Optika Microscope, Italy) were performed in order to phenotype identify the fungus. The process used to identify *A*. *quadrilineatus* at the molecular level was as follows: the fungal isolate cultured in sterile Petri plates using autoclaved Czapek’s yeast agar (CYA), and then it was incubated at 28°C for 7 days [24]. A Patho-gene-spin DNA/RNA extraction kit used to extract the DNA of the most potent fungus (*Aspergillus sp*.) and then transferred to Sol Gent Company in South Korea’s Daejeon for rRNA gene sequencing and PCR.

By ddNTPs in the reaction mixture, the PCR product (amplicons) was sequenced [25]. The sequences were analyzed using the BLAST algorithm from the National Centre of Biotechnology Information (NCBI) website. Phylogenetic analysis of the sequences was done using Meg Align (DNA Star) software version 5.05.


$$\;\%\;Deinking\;ability=\frac{A0-AT}{A0}\;\times 100$$ (1)

Where A0 = absorbance at zero time (control), and AT = absorbance after incubation. The maximum wavelength of toner black powder ink was 400 nm.

### Optimization of the deinking ability of *A*. *quadrilineatus*

50 mL of Bushnell and Hass broth medium was inoculated with one plug (8 mm in diameter) of an *A*. *quadrilineatus* culture that had been grown for seven days in an incubator at 28°C to examine the effects of various parameters (pH, temperature, ink initial concentration, and inoculum dose) on the ink elimination by *A*. *quadrilineatus*. All of the aforementioned tests were carried out in triplicate and incubated for 3, 6, and 9 days. Following incubation, the centrifugation of the fungus solution was at 6000 rpm for 10 min (Hettich Zentrifugen Mikro 22 R D-78532 Tuttlingen), and the supernatant was utilized with UV/Vis spectrophotometry (UV/Vis spectrophotometrically, Jasco-V 530) to measure the black toner powder ink solution’s content according to the equation (1).

### The temperature effect on the deinking ability of *A*. *quadrilineatus*

The temperature effect was studied by incubating cultures with 20000 mgL ⁻ ¹ black toner powder ink at 20, 25, 30, 35, and 40˚C.

### The hydrogen ion concentration effect on the deinking ability of A. *quadrilineatus*

The effect of pH: cultures were grown on media with 20000 mg L ⁻ ¹ ink adjusted with 1 mol L ⁻ ¹ HCl (aq) and NaOH (aq) to pH values of 2, 4, 6, 8, and 10.

### The initial ink concentration effect on deinking ability of *A*. *quadrilineatus*

Different concentrations of 5000, 10000, 20000, 30000, 40000, and 50000 mg/L were also created and utilized for culture to examine the influence of initial black toner powder ink concentration.

### The inoculum dose effect on the deinking ability of *A*. *quadrilineatus*

The effect of inoculum dose studied by inoculating 20000 mg/L of ink media with one, two, and three plugs (8 mm in diameter) of *A*. *quadrilineatus*.

### Scanning electron microscope (SEM) of *Aspergillus quadrilineatus* mycelia

The change of *Aspergillus quadrilineatus* growth after incubation in ink a liquid medium compared with its fungal growth on agar plate was observed using SEM, a scanning electron microscope (Quanta FEG 250, FEI, USA) at the Desert Research Centre in Cairo, Egypt used to observe the mycelium morphologies of this *A*. *quadrilineatus* in solution at an ink concentration of 20000 mg L ⁻ ¹ and on agar plates. SEM samples created according to [26] with the necessary adjustments.

### Study deinking of ink-loaded cellulose paper

This experiment aimed to explore the ability of *A*. *quadrilineatus* to eliminate the ink from ink-loaded filter paper. All optimized factors were applied, and a suspension of three mycelium plugs 8 mm in diameter of *A*. *quadrilineatus* in test tubes (9% NaCl w/v) was prepared. In this experiment, 9 cm diameter cellulose filter papers were first loaded with 5 mL black ink (20000 mg/L) and dried overnight, and then added into a Petri plate for inoculation with 5 mL of *A*. *quadrilineatus* suspension. After inoculation, they were incubated at 30°C for six days. Parallel testing was done using filter paper loaded with 5 mL of black ink without inoculation with 5 mL of *A*. *quadrilineatus* suspension (control) samples.

### Scanning electron microscopy of ink-loaded cellulose filter paper

In this experiment, the ink-loaded cellulose paper before and after treatment with 5 ml of *A*. *quadrilineatus* suspension was washed thrice with deionized water and was gradually dehydrated with an acetone gradient between 30% and 90% and finally suspended in 100% acetone; small pieces of fibers were air dried and placed on the stubs, mounted with silver tape, and sputter coated with gold using fine coat (Quanta FEG 250, FEl, USA) and examined.

### FT-IR analysis of ink-loaded cellulose filter paper

After studying the deinking ability of *A*. *quadrilineatus* suspension for ink-loaded cellulose filter paper, FT-IR analysis analyzed the ink-loaded filter paper before and after treatment with 5 mL of *A*. *quadrilineatus* suspension (Perkin-Elmer Spectrum version 10.5.4 at the central lab, Faculty of Science, Helwan University, and Cairo, Egypt). The FT-IR spectra have a resolution of 4 cm^-1^ and a wavenumber range of 400–4000 cm^-1^.

### The mechanism of the ink elimination by *A. quadrilineatus*

The ink adsorption by *A. quadrilineatus* was modeled using the Langmuir and Freundlich adsorption isotherms (eq. 2 and eq. 3) at 5000–50000 mg/L^-1^ ink concentrations, pH 6, 30°C, and 4.0 g/L^-1^ fungal biomass.

They stated:


$$qe=\frac{qmKLCe}{1+KLCe}$$ (2)

$$qe=KFCe\frac{1}{n}\;$$ (3)

Where KL is the Langmuir equilibrium parameter (L mg^-1^) and represents the affinity at the binding site, qe is the equilibrium adsorption capacity (mg g^-1^), Ce is the concentration of ink in the solution at equilibrium (mg l^-1^), and KF and n are Freundlich constants, representing the adsorption capacity and adsorption strength, respectively.

### Screening the enzyme activity of *A*. *quadrilineatus*

This experiment aimed to explore the mechanism of black ink eliminated by *A*. *quadrilineatus* via screening its ability for xylanase and lipase production.

*A*. *quadrilineatus* was grown on substrate agar plates containing (g/L): 0.5g NaCl, 1g KH_2_PO_4_ MnSO_4_, 0.3g NH_4_ NO_3_, 0.5g MgSO_4_, 0.01g FeSO_4_, and 6 g agar supplemented with 1% (w/v) beech wood xylan.

After *A*. *quadrilineatus* was inoculated at the plate center, it was incubated for five days at 30°C. It was then stained with 0.1% Congo red for ten minutes to observe and measure halo diameters for xylanase activity [27]. And also, preliminary screening of *A*. *quadrilineatus* for lipase production was performed by the plate-based screening method. This was carried out by inoculating the different isolates on Tween 80 agar plates and incubating at 28°C for 5 days.

The lipase-producing *A*. *quadrilineatus* showed an opalescent zone around the growth [28].

### Ethics approval and consent to participate

Not applicable.

### Statistical analysis

The data are shown as mean ± standard deviation (SD). Version 22.0 of SPSS software was used for all statistical analyses.

## Results

### Screening and identification methods

**Fig 2:**
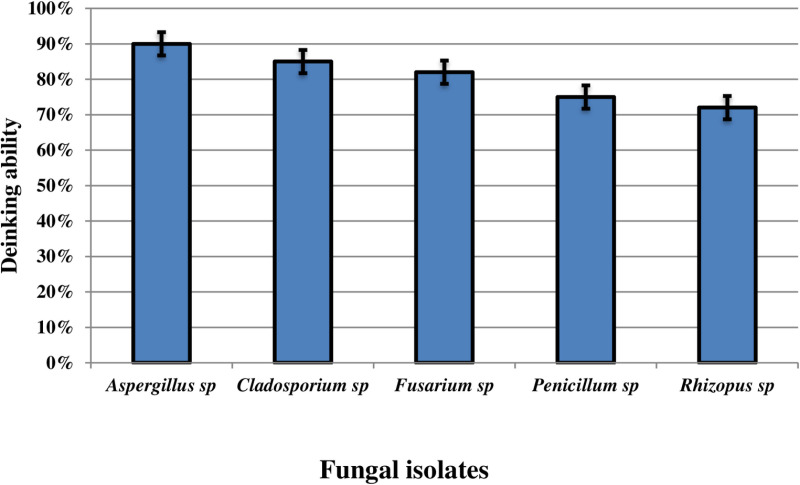
Screening deinking ability of five fungal isolates.

**Fig 3:**
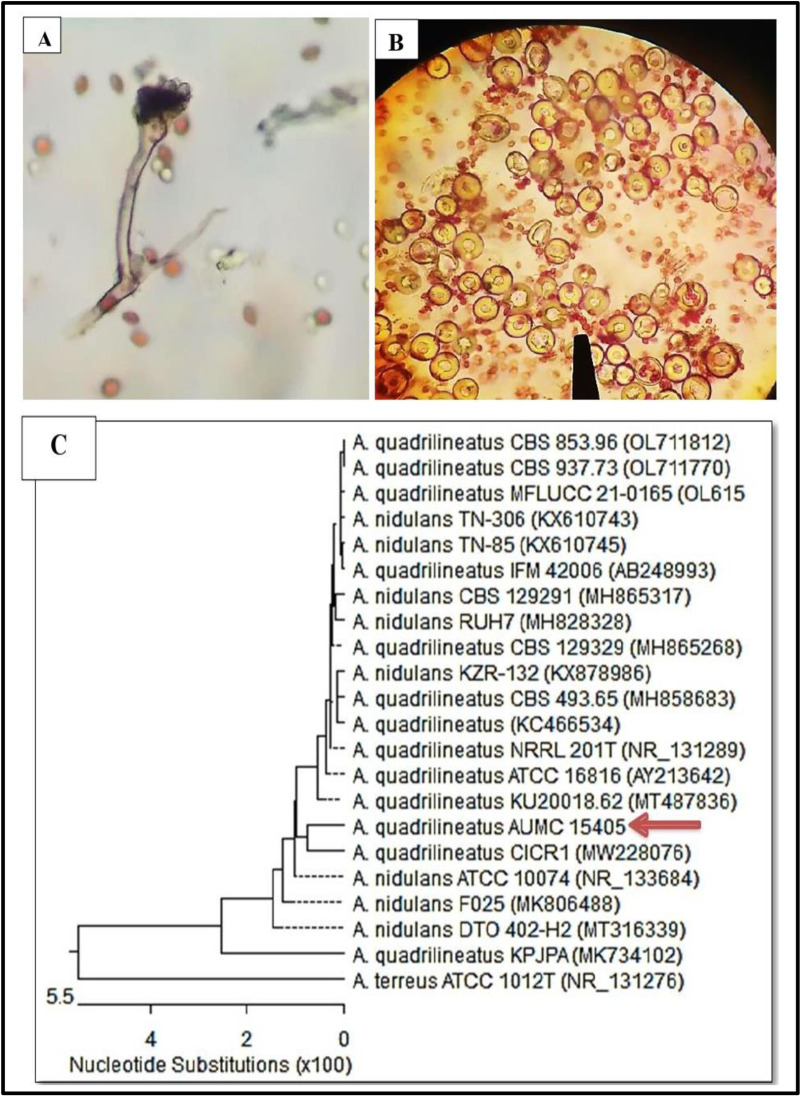
*Aspergillus quadrilineatus* (*Emericella quadrilineata*). **(A)** Coniophore and conidia. **(B)** Hülle cells and conidia (Optika Microscope, Italy) at a magnification of 40X. **(C)** A phylogenetic tree that is aligned with strains from Gen Bank that are closely related to *Aspergillus quadrilineatus*.

Using Bushnell and Hass media, five fungal isolates extracted from soil contaminated by ink traces. The isolates were *Aspergillus sp*., *Cladosporium sp*., *Fusarium sp*., *Penicillium sp*., and *Rhizopus sp*. The results showed that the fungal isolate named *Aspergillus sp*. achieved the highest deinking ability (percentage) among the five fungal isolates (Fig 2), with a deinking rate of 90%. The identification of *Aspergillus sp* based on the external features of the strain. *Aspergillus sp* was preliminarily identified as *A*. *quadrilineatus*, (Fig 3A and 3B). The fungal conidia were spherical, smooth-walled, sub-hyaline, and finely roughened. After 3 weeks of incubation, purple ascocarps formed, surrounded by characteristic hülle cells.

The ascospores were reddish in color, and smooth surface. Then, the identification was confirmed by phylogenetic analysis, in which the BLAST search of ITS rRNA sequencing results of *A*. *quadrilineatus* was performed in the NCBI database, and the phylogenetic tree was plotted. As seen from (Fig 3C), strain *A*. *quadrilineatus* had a 100% similarity with *A*. *quadrilineatus* AUMC 15405.

### Optimization of the deinking ability of *A*. *quadrilineatus*

#### Temperature effect.

**Fig 4:**
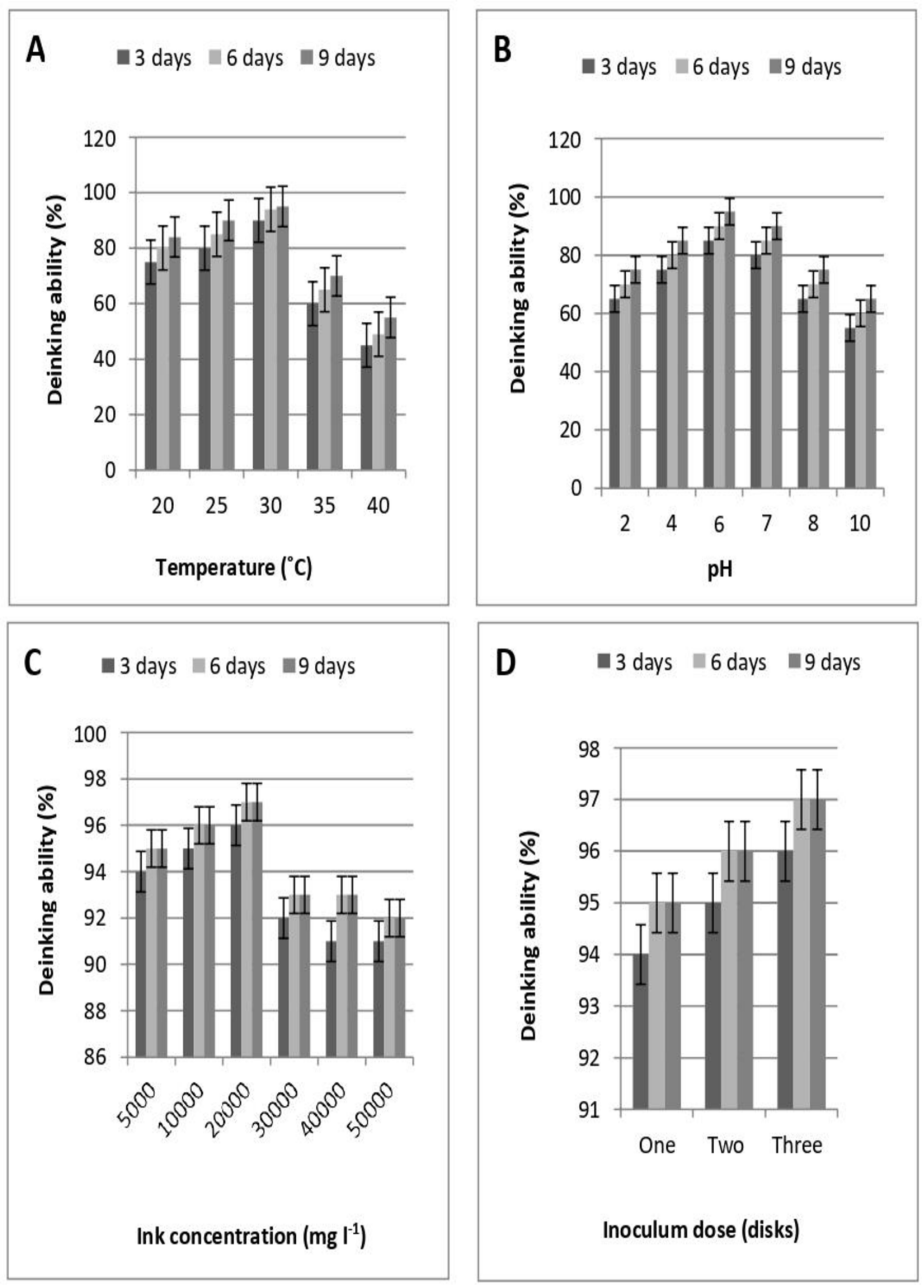
Factors that affect the deinking ability of *A*. *quadrilineatus*. (A) Temperature. (B) pH. (C) Ink concentrations. (D) Inoculum dose.

As can be seen from (Fig 4A), within the temperature range of 20–40˚C, the deinking percentage by *A*. *quadrilineatus* increased with increasing temperature and reached a peak value (94%) at 30˚C after 9 days of incubation time.

Afterward, as the temperature increased, the percentage of ink removal achieved by *A*. *quadrilineatus* gradually decreased.

#### pH effect.

(Fig 4B) shows that *A*. *quadrilineatus* successfully used a wide range of pH 2–10, confirming the adaptability of *A*. *quadrilineatus* to the pH stress.

When the initial pH of the solution was in the range of 2–6, the deinking percentage of *A*. *quadrilineatus* increased significantly with increasing pH and reached a peak value of 93% at pH = 6 after 9 days of incubation time. When the initial solution pH was 6–10, the ink removal extent by *A*. *quadrilineatus* decreased with increasing pH.

#### The initial ink concentration effect.

The findings in (Fig 4C) show that as the ink concentration in the system gradually increased there was an evident decline trend in the amount of ink removed by *A*. *quadrilineatus*. *A*. *quadrilineatus* removed up to 97% of the ink when the ink concentration was between 5000 and 20000 mg L^-1^. *A*. *quadrilineatus* significantly reduced ink removal when ink concentration exceeded 20000 mg L^-1^.

#### The inoculum dose effect.

The results presented in (Fig 4D) show that the ink removal increased substantially as the inoculum dose increased.

The most efficient ink removal rate in liquid media in this study was 97% after 6 days of incubation time, achieved by using an inoculum dose in three fungal discs.

### Scanning electron microscope (SEM) of *A*. *quadrilineatus*

**Fig 5:**
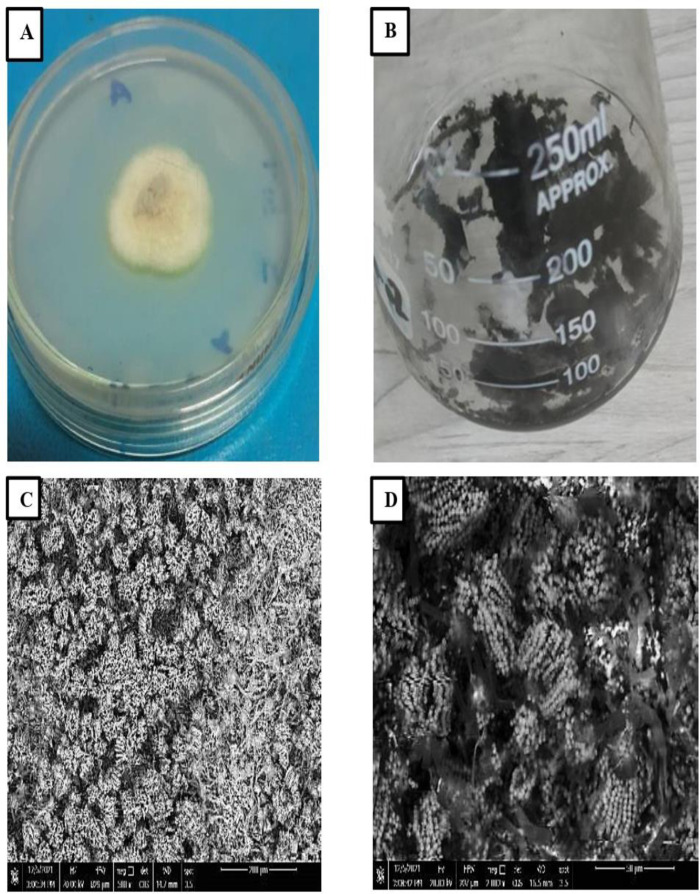
Scanning electron microscope profile *A*. *quadrilineatus* filament morphology. **(A)** *A*. *quadrilineatus* growth on agar plate. **(B)** Fifty mL of ink solution inoculated with *A*. *quadrilineatus*, after 6 days incubation. **(C)** Scanning control with zero ink concentration. **(D)** Scanning *A*. *quadrilineatus* treated with ink concentration 20000 mgL^-1^. The magnification is 2000x.

Using SEM photomicrographs of *A*. *quadrilineatus* at magnification powers (2000×) showed the morphological changes on the *A*. *quadrilineatus* surface before and after ink elimination (Fig 5A-5D). Hyphae surfaces at zero ink concentration are normal surfaces with no attached ink on the surfaces; also noticed the formation of conidia (Fig 5C). However, at 20000 mg/L ink concentration, *A*. *quadrilineatus* surfaces with changes in hyphae surfaces were observed to have more dark-colored hyphae due to the attachment of ink to hyphae surfaces and reduced conidia formation compared to zero ink concentration (Fig 5D). Also, the pores on the surface of *A*. *quadrilineatus* became invisible because the external biomass surface had been covered by the superficial ink.

### Study deinking of ink-loaded cellulose paper

**Fig 6:**
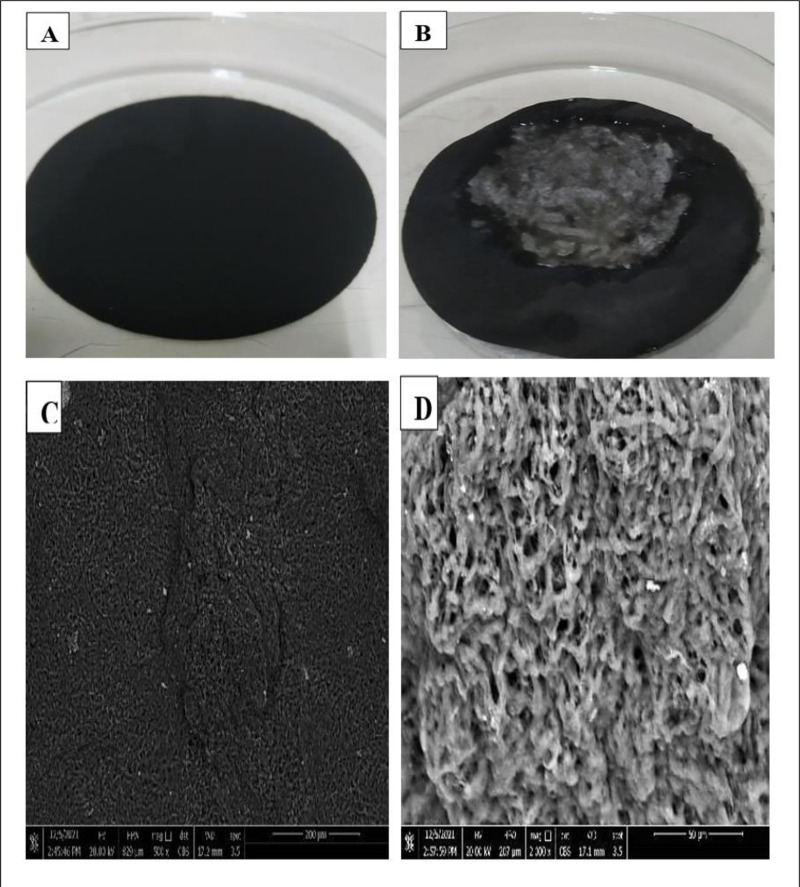
Deinking and Scanning electron microscope profile ink-loaded cellulose filter paper. **(A)** Filter paper with 5 mL black ink (20000 mg/L). **(B)** The treated ink-loaded cellulose papers with 5 mL of *A*. *quadrilineatus* **(C)** Scanning the filter paper with ink concentration 20000 mgL^-1^. **(D)** Scanning the treated ink-loaded cellulose paper with 5 mL of *A*. *quadrilineatus*. The magnification is 2000x.

The results in (Fig 6) show the ability of *A. quadrilineatus* to eliminate the ink particles from the surface of the ink-loaded filter paper and prevent deposition onto the fibre surfaces, as in (Fig 6B), which was treated with 5 mL of fungal suspension, in contrast to the untreated paper (control), as in (Fig 6A).

### Scanning electron microscopy of ink-loaded cellulose filter paper

Filter paper surface morphologies both before and after treatment by 5 mL of *A. quadrilineatus* were observed by SEM as shown in Fig 6C and 6D. A surface with modifications was seen adsorpting the ink in which the ink particles were uniformly deposited onto the filter paper surface (Fig 6C).

However, the filter paper surface morphology had changed into a rough surface without any attachments of the ink deposition (Fig 6D). These results show the strong deinking ability of *A. quadrilineatus* suspension for removing ink from ink-loaded filter papers.

### FT-IR analysis of ink-loaded cellulose filter paper

**Fig 7:**
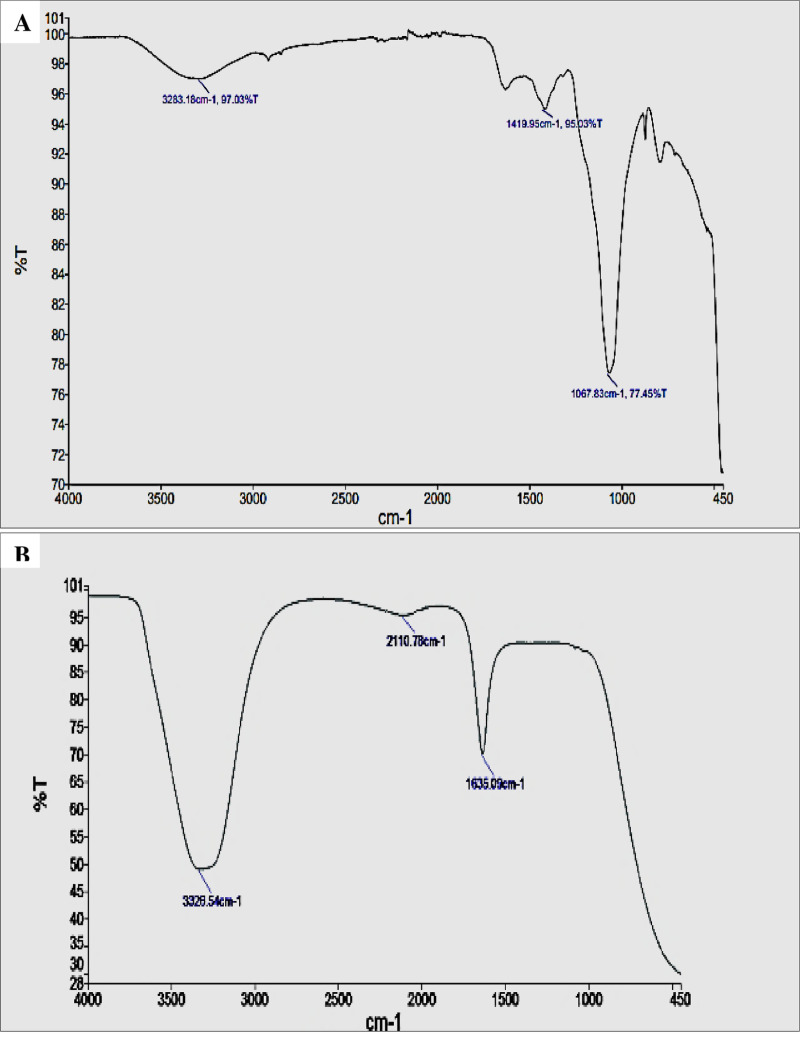
FT-IR ink-loaded cellulose filter paper morphology. **(A)** Control with ink concentration 20000 mgL^-1^. **(B)** The treated ink-loaded cellulose paper with 5 mL of *A*. *quadrilineatus*.

FT-IR absorption peaks in Fig 7 were assigned according to the literature [29]. FT-IR analysis of the ink-loaded cellulose filter paper (before and after treatment) produced different peaks to identify the functional groups of control and treatments. For the control (before ink elimination) spectrum as shown in Fig 7A, the absorption bands were assigned at 3326.54 cm^-1^ and attributed to hydroxyl groups, at 2110.78 cm^-1^ and attributed to the allene, ketene, isocyanate, and isothiocyanate groups, and at 1635.09 cm^-1^ which is ascribed to the bend of the amide group. While, after the treatment (after ink elimination) of ink-loaded filter paper by *A. quadrilineatus*, as shown in Fig 7B, gave a band at 3283.18 cm^-1^, a band at 1419.95 cm^-1^, a band at 1069.83 cm^-1^.

### The mechanism of the black ink elimination by *A. quadrilineatus*

**Fig 8:**
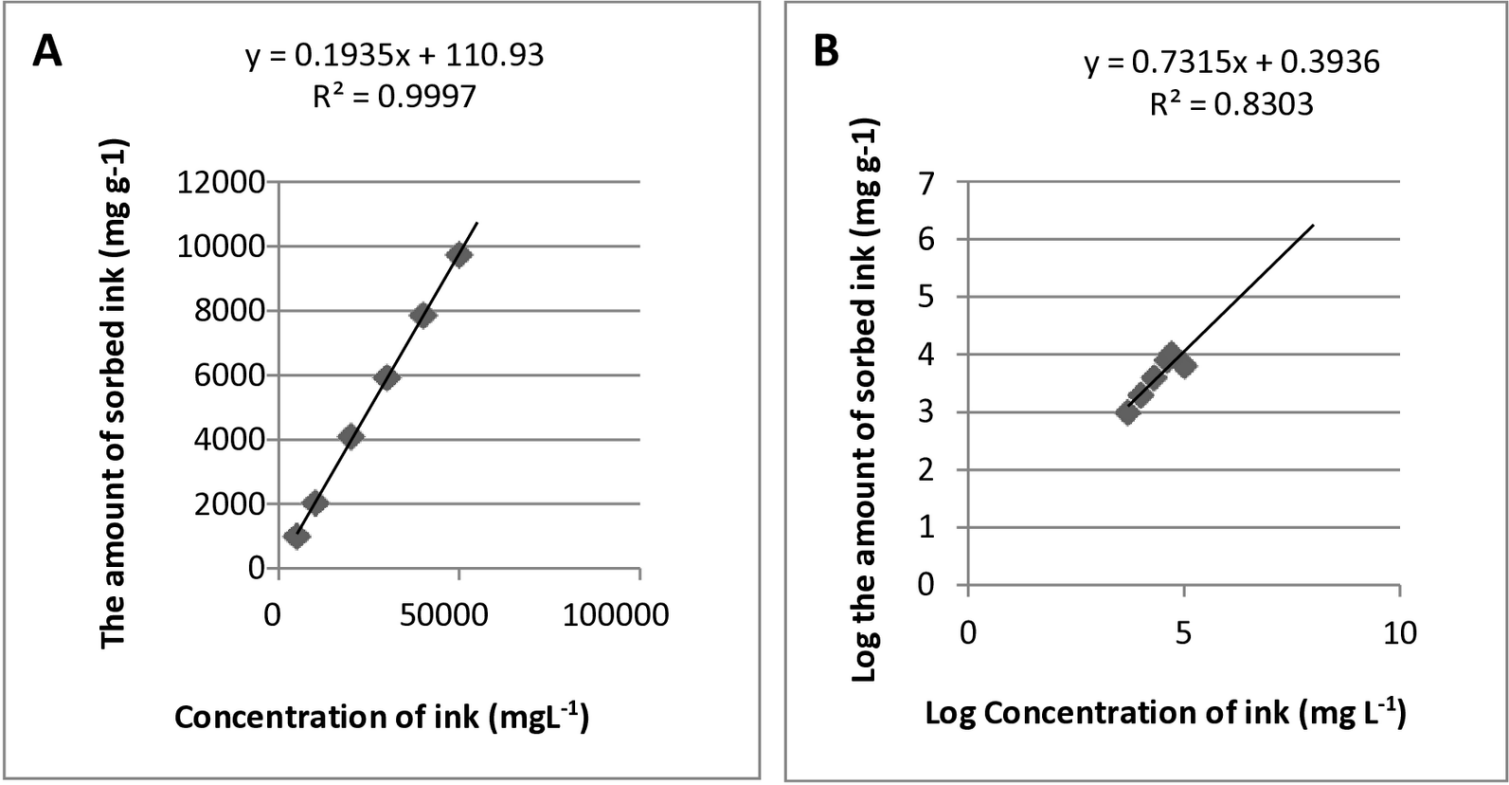
The adsorption isotherm model of *A*. *quadrilineatus.* **(A)** Langmuir model. **(B)** Freundlich model.

Langmuir’s and Freundlich’s adsorption isotherm models were used to simulate the adsorption of ink by *A. quadrilineatus* in this work to forecast biosorption behaviour and determine the biosorption capacity. The *A. quadrilineatus* adsorption isotherm model is shown in Fig 8A and 8B. The outcomes revealed that when ink concentration increased, *A. quadrilineatus*’s ability for ink adsorption appeared to trend upward before achieving equilibrium in a saturated state. The two adsorption models’ correlation coefficient comparisons revealed that the adsorption process was a good fit for the Langmuir equation, showing that the ink adsorption by *A. quadrilineatus* is a monolayer adsorption process dominated by surface adsorption.

### Screening the enzyme activity of *A. quadrilineatus*

**Fig 9:**
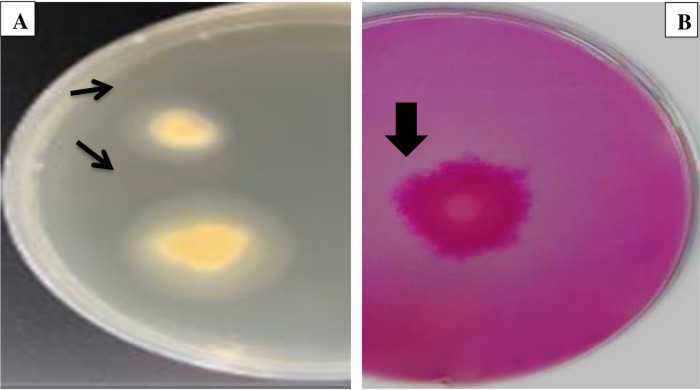
Screening the enzyme activity of *A*. *quadrilineatus.* **(A)** Lipase enzyme. **(B)** Xylanase enzyme on Tween 80 agar medium. Arrows indicate opalescent zone around the fungal growth and, the faint red in color, respectively.

(Fig 9)‘s findings demonstrated the capacity of *A. quadrilineatus* to produce lipase and xylanase enzymes on agar plates. (Fig 9A) confirmed the ability of *A. quadrilineatus* to produce the lipase enzyme on Tween 80 agar medium by the formation of an opalescent zone around the fungal growth. Moreover, examination of xylanase production by *A. quadrilineatus* was confirmed by the clear zone formation around the fungal inoculum on xylan agar plates due to hydrolysis; Congo red-stained xylan medium thus appeared faint red in color, as shown in Fig 9B.

## Discussion

The mycoremediation has improved over the past few decades, and still important to consider when selecting such an organism. In the present study five filamentous fungal isolates obtained from ink-contaminated soil and screened for their deinking ability under submerged fermentation. The most effective fungus for the elimination of black ink in this investigation was *A. quadrilineatus,* which identified molecularly as *A. quadrilineatus* AUMC15405.

These might bring on by the molecular sizes and ink’s chemical composition.

The optimization experiments confirmed that 30°C was the greatest temperature at which the highest deinking (94%) could be obtained after an incubation period of 9 days. The temperature has a favorable impact on deinking, according to Lech et al. [30], who also noted that the efficacy of biosorption decreases at higher temperatures. Furthermore the biosorption effectiveness was only reduced by 15% to 50% when the temperature rose.

Additionally, it was determined that at pH 6, *A. quadrilineatus* obtained 93% deinking after 9 days of incubation, according to our research on the impact of pH on the removal of black ink. The degree of ink removal by *A. quadrilineatus* reduced with rising pH, when the pH of the solution was in the range of 6–10, indicating that *A. quadrilineatus* has a high H⁺ tolerance. The present results are in line with those of Sun et al. [31], who reported that the best Sb (V) biosorption efficiency by Microcystis was obtained at pH 2.8 and that a higher pH value caused increased repulsion between anionic Sb (V) and biomass surface and decreased the biosorption.

In the current investigation, *A. quadrilineatus* removed up to 97% of the ink when the concentration was in the range of 5000–20,000 mgL ⁻ ¹. *A. quadrilineatus* significantly reduced ink removal when ink concentration exceeded 20000 mg/L. According to Li et al. [32], high ink concentrations had a substantial toxic effect on cell development and cell membrane function, and they also discovered that fungal cell proliferation and their capacity to absorb ink were hindered. Our results support their findings. The outcomes revealed that the ink removal capacity of *A. quadrilineatus* significantly reduced when ink concentration increased. Also *A. quadrilineatus* displayed an apparent upward trend with increasing ink concentration before achieving equilibrium in a saturated state. As indicated earlier by Abdolali et al. [33], this linked to an increase in the likelihood of contact between adsorbate and mycelia. Moreover, the current investigation showed that *A. quadrilineatus*’s ability to biosorb ink depended on the inoculum dose. Using an inoculum dose in three fungal discs was effective in ink removal rate in liquid media, which achieved 97% of deinking after 6 days of incubation. This may be because a higher inoculum dose of fungal cells will result in more active sites being provided by the fungal cells for eliminating the ink, giving more opportunities for the fungal cell surface to contact and bind with ink when the mass concentration of ink is constant and the adsorption sites on the cell surface are not saturated.

Consequently a higher ink removal capacity was attained as a result. Amirnia et al. [34] revealed that increasing the inoculum dose of fungi boosted the mutual adsorption between fungi. We learnt the ink particles uniformly deposited onto smooth fungal filaments via SEM pictures. These findings demonstrate the fungal filaments’ affinity for removing ink from liquids and congruent with the findings of Shuhui et al. [35], who found that *A. niger*’s cell surface properties began to change in the presence of acid anionic dyes in the culture medium and were successful in removing dyes from wastewater.

*A. quadrilineatus* suspension successfully resists deposition onto the fibre surfaces during studying the deinking of treated and untreated (control) ink-loaded cellulose filter paper. The differences in morphology and functional groups before and after the black ink elimination were determined using SEM and FT-IR studies. These findings demonstrate the fungal suspension’s affinity for removing ink from ink-loaded cellulose filter paper. Additionally, the FT-IR results showed that hydroxyl, amide, and sulfoxide would be the primary functional groups involved in the treatment of ink-loaded cellulose filter paper by 5 mL of *A. quadrilineatus*’s suspension. This suggests that there were interactions between the ink and these functional groups and that these same functional groups were also responsible for the ink removal. On the control spectrum, some of these groups were present. There was a difference in function groups between the treated and untreated ink-loaded cellulose filter paper, according to FT-IR analyses. The two adsorption models’ correlation coefficient comparisons revealed that the adsorption process suited the Langmuir equation well, suggesting that *A. quadrilineatus*’s ink adsorption is a monolayer adsorption process dominated by surface adsorption. The qualitative substrate agar test indicated positive results, where the xylanase-producing test displayed clearing zones from a dark red to a light red color representing efficient xylanase activity. Pretreatment of ONP pulp with xylanase obtained a brightness of 59.6 ± 0.8% ISO, which was 8.5, 11.4 and 21.6% higher than that of the P-L-C, P-X-C and untreated pulp [36]. In the case of the lipase enzyme production test, an opalescent zone was displayed. Many oils have been used as carbon sources to produce lipase enzymes from microbial strains, such as olive oil, sunflower oil, almond oil, and palm oil [37]. Enzymes such as lipase and xylanase used as, substitutes for the conventional chemical methods of deinking waste paper [38,39]. The results of SEM, FTIR analyses, Langmuir and Freundlich adsorption isotherms, and qualitative screening of xylanase and lipase enzyme production indicated that adsorption could be the primary method of color reduction for viable *A*. *quadrilineatus* in which the ink was seen to be adsorbed on *A*. *quadrilineatus* mycelia initially, and as a result of the enzymatic degradation, the dye’s color was eliminated from the culture as well as from the cellulose filter paper’s surface.

## Conclusions

Under numerous optimization variables, including temperature 30, pH 6, beginning ink concentrations of 20,000 mg/L, and inoculum dose of 3 fungal discs, *Aspergillus quadrilineatus*, which was isolated from a soil contaminated by ink residues, has achieved a 97% clearance percentage of black toner ink.

The deinking mechanism of *A. quadrilineatus* has been observed using, SEM and FTIR analyses, Langmuir and Freundlich adsorption isotherms, and qualitative screening of xylanase and lipase enzymes production.

*A. quadrilineatus* also has a discernible impact on the removal of ink from cellulose paper that has been loaded with ink. More evidence in favor of the practical application of ink removal through wastewater and waste papers needs to be obtained through further study.

## Supporting information

S1 Fig Data obtained from molecular identification analysis of *Asperrgillus quadrilineatus* and used to build a phylogenetic tree as mentioned in Fig 3C (A portion of the data was used in the reported study). (PDF)

S1 Table The values used to build graph and SD values of Fig 2. (PDF)

S2 Table The values used to build graph and SD values of Fig 4A-4D. (PDF)
