# Supplementary material for: The removal of black ink via Emericella quadrilineata as a green alternative technique to recycling ink waste papers
Source: PLoS One. 2025 May 29;20(5):e0324022. doi: 10.1371/journal.pone.0324022 (PMC12122025; doi:10.1371/journal.pone.0324022)
Supplement: S1 Fig — (PDF) [file pone.0324022.s001.pdf]

Sample: *Asperrgillus quadrilineatus* AUMC15405 (553 letters)

AGGTGAAAACTGCGGAAGGATCATTACCGAGTGCGGGCTGCCTCCGGGCGCCCAACCTCCCACCCGTGACTACCTAACACTGTTGCTTCGGCGGGGAGCCCCCT
AGGGGCGAGCCGCCGGGGACCACTGAACTTCATGCCTGAGAGTGATGCAGTCTGAGCCTGAATACAAATCAGTCAAAACTTTCAACAATGGATCTCTTGGTTCC
GGCATCGATGAAGAACGCAGCGAACTGCGATAAGTAATGTGAATTGCAGAATTCAGTGAATCATCGAGTCTTTGAACGCACATTGCGCCCCCTGGCATTCCGGG
GGGCATGCCTGTCCGAGCGTCATTGCTGCCCTCAAGCCCGGCTTGTGTGTTGGGTCGTCGTCCCCCCCGGGGGACGGGCCCGAAAGGCAGCGGCGGCACCGTGT
CCGGTCCTCGAGCGTATGGGGCTTTGTCACCCGCTCGATTAGGGCCGGCCGGGCGCCAGCCGGCGTCTCCAACCTTATTTTTCTCAGGTTGACCTCGGATCAGGT
AGGGATACCCGCTGAACTTAAGCATATCAATA

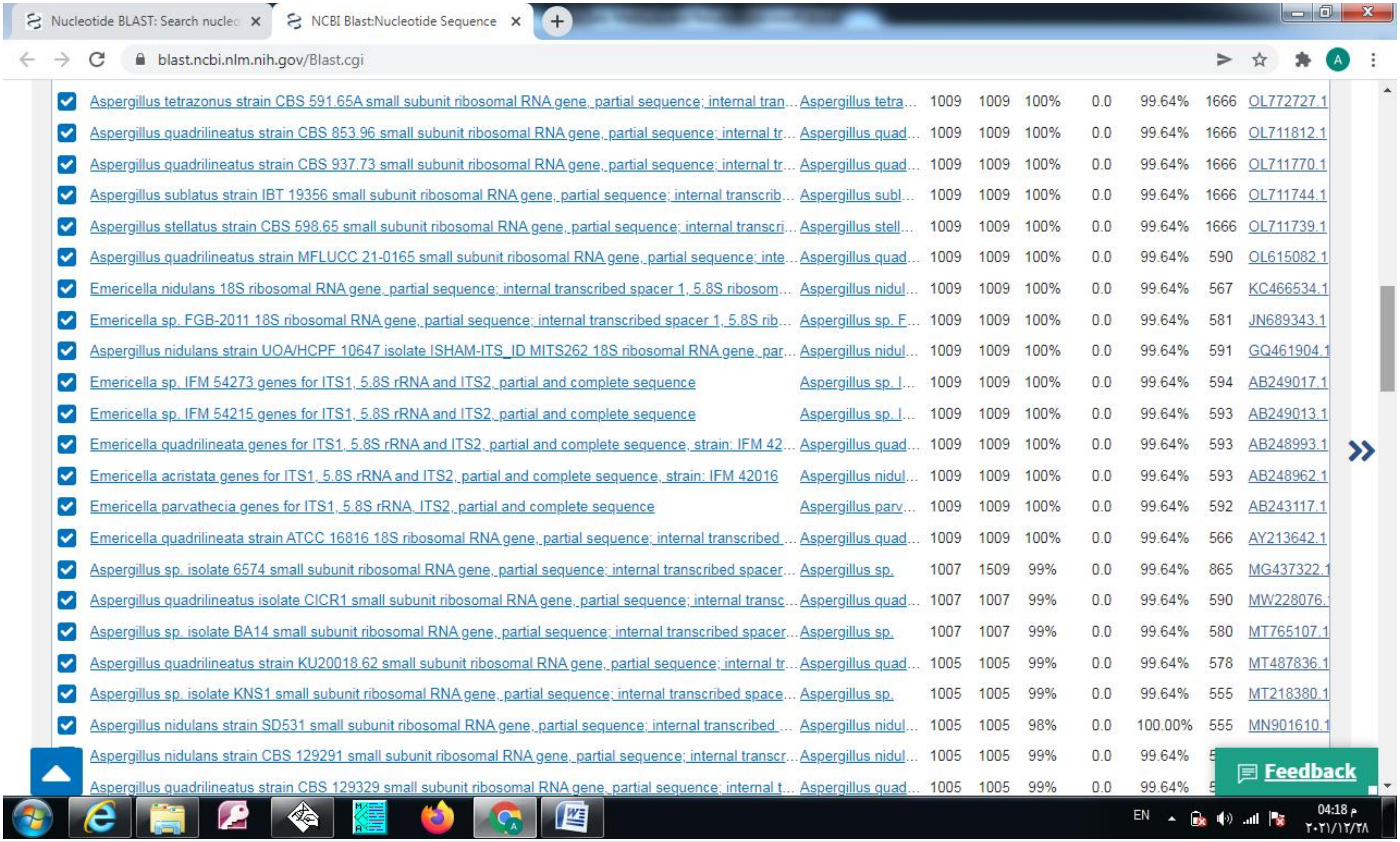

Aspergillus tetrazonus strain CBS 591.65A small subunit ribosomal RNA gene, partial sequence; internal tran...	Aspergillus tetra...	1009	1009	100%	0.0	99.64%	1666	OL772727.1
Aspergillus quadrilineatus strain CBS 853.96 small subunit ribosomal RNA gene, partial sequence; internal tr...	Aspergillus quad...	1009	1009	100%	0.0	99.64%	1666	OL711812.1
Aspergillus quadrilineatus strain CBS 937.73 small subunit ribosomal RNA gene, partial sequence; internal tr...	Aspergillus quad...	1009	1009	100%	0.0	99.64%	1666	OL711770.1
Aspergillus sublatus strain IBT 19356 small subunit ribosomal RNA gene, partial sequence; internal transcrib...	Aspergillus subl...	1009	1009	100%	0.0	99.64%	1666	OL711744.1
Aspergillus stellatus strain CBS 598.65 small subunit ribosomal RNA gene, partial sequence; internal transcri...	Aspergillus stell...	1009	1009	100%	0.0	99.64%	1666	OL711739.1
Aspergillus quadrilineatus strain MFLUCC 21-0165 small subunit ribosomal RNA gene, partial sequence; inte...	Aspergillus quad...	1009	1009	100%	0.0	99.64%	590	OL615082.1
Emericella nidulans 18S ribosomal RNA gene, partial sequence; internal transcribed spacer 1, 5.8S ribosom...	Aspergillus nidul...	1009	1009	100%	0.0	99.64%	567	KC466534.1
Emericella sp. FGB-2011 18S ribosomal RNA gene, partial sequence; internal transcribed spacer 1, 5.8S rib...	Aspergillus sp. F...	1009	1009	100%	0.0	99.64%	581	JN689343.1
Aspergillus nidulans strain UOA/HCPF 10647 isolate ISHAM-ITS_ID MITS262 18S ribosomal RNA gene, par...	Aspergillus nidul...	1009	1009	100%	0.0	99.64%	591	GQ461904.1
Emericella sp. IFM 54273 genes for ITS1, 5.8S rRNA and ITS2, partial and complete sequence	Aspergillus sp. I...	1009	1009	100%	0.0	99.64%	594	AB249017.1
Emericella sp. IFM 54215 genes for ITS1, 5.8S rRNA and ITS2, partial and complete sequence	Aspergillus sp. I...	1009	1009	100%	0.0	99.64%	593	AB249013.1
Emericella quadrilineata genes for ITS1, 5.8S rRNA and ITS2, partial and complete sequence, strain: IFM 42...	Aspergillus quad...	1009	1009	100%	0.0	99.64%	593	AB248993.1
Emericella acristata genes for ITS1, 5.8S rRNA and ITS2, partial and complete sequence, strain: IFM 42016	Aspergillus nidul...	1009	1009	100%	0.0	99.64%	593	AB248962.1
Emericella parvathecia genes for ITS1, 5.8S rRNA, ITS2, partial and complete sequence	Aspergillus parv...	1009	1009	100%	0.0	99.64%	592	AB243117.1
Emericella quadrilineata strain ATCC 16816 18S ribosomal RNA gene, partial sequence; internal transcribed ...	Aspergillus quad...	1009	1009	100%	0.0	99.64%	566	AY213642.1
Aspergillus sp. isolate 6574 small subunit ribosomal RNA gene, partial sequence; internal transcribed spacer...	Aspergillus sp.	1007	1509	99%	0.0	99.64%	865	MG437322.1
Aspergillus quadrilineatus isolate CICR1 small subunit ribosomal RNA gene, partial sequence; internal transc...	Aspergillus quad...	1007	1007	99%	0.0	99.64%	590	MW228076.[illegible]
Aspergillus sp. isolate BA14 small subunit ribosomal RNA gene, partial sequence; internal transcribed spacer...	Aspergillus sp.	1007	1007	99%	0.0	99.64%	580	MT765107.1
Aspergillus quadrilineatus strain KU20018.62 small subunit ribosomal RNA gene, partial sequence; internal tr...	Aspergillus quad...	1005	1005	99%	0.0	99.64%	578	MT487836.1
Aspergillus sp. isolate KNS1 small subunit ribosomal RNA gene, partial sequence; internal transcribed space...	Aspergillus sp.	1005	1005	99%	0.0	99.64%	555	MT218380.1
Aspergillus nidulans strain SD531 small subunit ribosomal RNA gene, partial sequence; internal transcribed ...	Aspergillus nidul...	1005	1005	98%	0.0	100.00%	555	MN901610.1
Aspergillus nidulans strain CBS 129291 small subunit ribosomal RNA gene, partial sequence; internal transcr...	Aspergillus nidul...	1005	1005	99%	0.0	99.64%	[illegible]	[illegible]
Aspergillus quadrilineatus strain CBS 129329 small subunit ribosomal RNA gene, partial sequence; internal t...	Aspergillus quad...	1005	1005	99%	0.0	99.64%	[illegible]	[illegible]

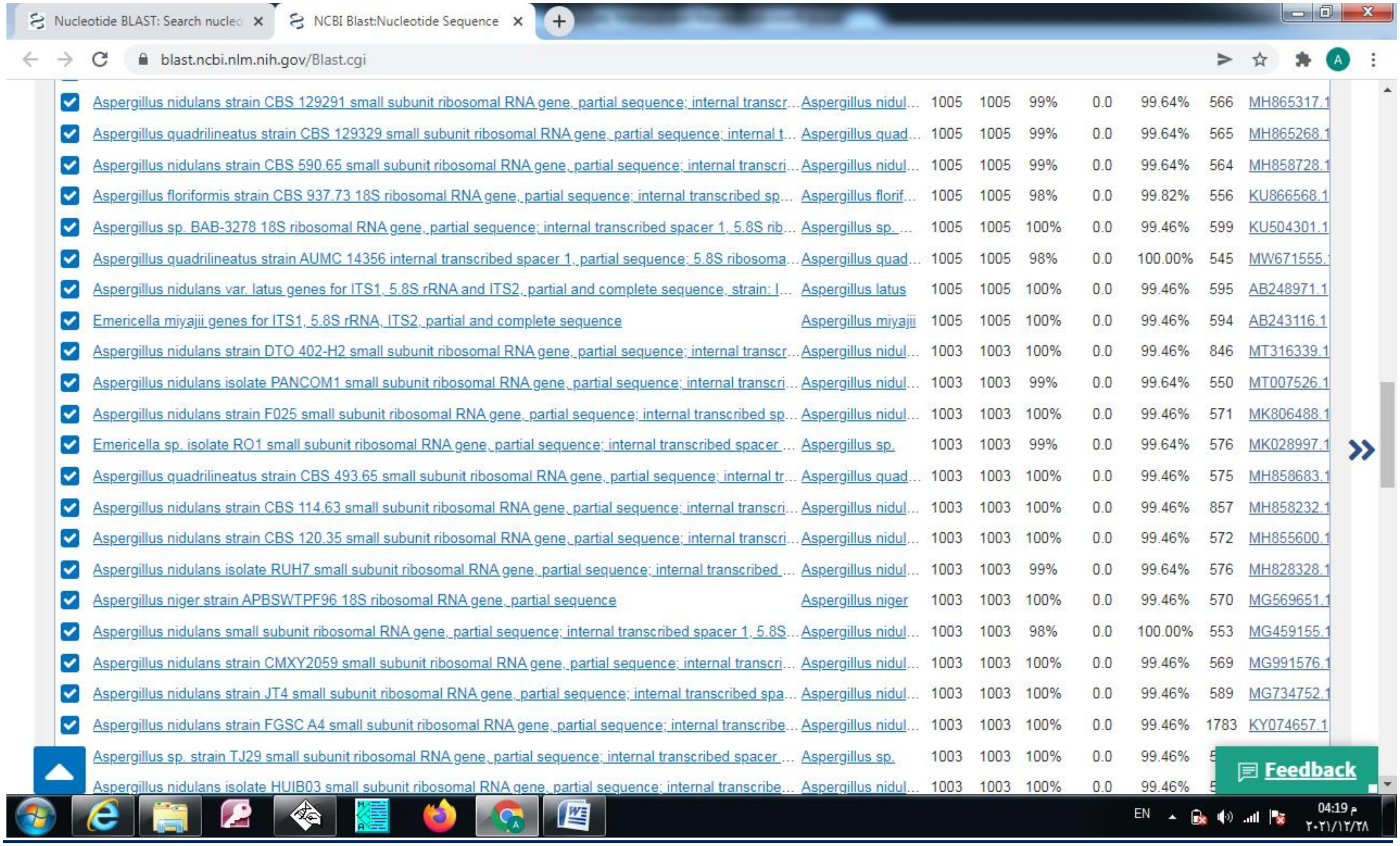

Aspergillus nidulans strain CBS 129291 small subunit ribosomal RNA gene, partial sequence; internal transcr...	Aspergillus nidul...	1005	1005	99%	0.0	99.64%	566	MH865317.1
Aspergillus quadrilineatus strain CBS 129329 small subunit ribosomal RNA gene, partial sequence; internal t...	Aspergillus quad...	1005	1005	99%	0.0	99.64%	565	MH865268.1
Aspergillus nidulans strain CBS 590.65 small subunit ribosomal RNA gene, partial sequence; internal transcri...	Aspergillus nidul...	1005	1005	99%	0.0	99.64%	564	MH858728.1
Aspergillus floriformis strain CBS 937.73 18S ribosomal RNA gene, partial sequence; internal transcribed sp...	Aspergillus florif...	1005	1005	98%	0.0	99.82%	556	KU866568.1
Aspergillus sp. BAB-3278 18S ribosomal RNA gene, partial sequence; internal transcribed spacer 1, 5.8S rib...	Aspergillus sp. ...	1005	1005	100%	0.0	99.46%	599	KU504301.1
Aspergillus quadrilineatus strain AUMC 14356 internal transcribed spacer 1, partial sequence; 5.8S ribosoma...	Aspergillus quad...	1005	1005	98%	0.0	100.00%	545	MW671555.[illegible]
Aspergillus nidulans var. latus genes for ITS1, 5.8S rRNA and ITS2, partial and complete sequence, strain: I...	Aspergillus latus	1005	1005	100%	0.0	99.46%	595	AB248971.1
Emericella miyajii genes for ITS1, 5.8S rRNA, ITS2, partial and complete sequence	Aspergillus miyajii	1005	1005	100%	0.0	99.46%	594	AB243116.1
Aspergillus nidulans strain DTO 402-H2 small subunit ribosomal RNA gene, partial sequence; internal transcr...	Aspergillus nidul...	1003	1003	100%	0.0	99.46%	846	MT316339.1
Aspergillus nidulans isolate PANCOM1 small subunit ribosomal RNA gene, partial sequence; internal transcri...	Aspergillus nidul...	1003	1003	99%	0.0	99.64%	550	MT007526.1
Aspergillus nidulans strain F025 small subunit ribosomal RNA gene, partial sequence; internal transcribed sp...	Aspergillus nidul...	1003	1003	100%	0.0	99.46%	571	MK806488.1
Emericella sp. isolate RO1 small subunit ribosomal RNA gene, partial sequence; internal transcribed spacer ...	Aspergillus sp.	1003	1003	99%	0.0	99.64%	576	MK028997.1
Aspergillus quadrilineatus strain CBS 493.65 small subunit ribosomal RNA gene, partial sequence; internal tr...	Aspergillus quad...	1003	1003	100%	0.0	99.46%	575	MH858683.1
Aspergillus nidulans strain CBS 114.63 small subunit ribosomal RNA gene, partial sequence; internal transcri...	Aspergillus nidul...	1003	1003	100%	0.0	99.46%	857	MH858232.1
Aspergillus nidulans strain CBS 120.35 small subunit ribosomal RNA gene, partial sequence; internal transcri...	Aspergillus nidul...	1003	1003	100%	0.0	99.46%	572	MH855600.1
Aspergillus nidulans isolate RUH7 small subunit ribosomal RNA gene, partial sequence; internal transcribed ...	Aspergillus nidul...	1003	1003	99%	0.0	99.64%	576	MH828328.1
Aspergillus niger strain APBSWTPF96 18S ribosomal RNA gene, partial sequence	Aspergillus niger	1003	1003	100%	0.0	99.46%	570	MG569651.1
Aspergillus nidulans small subunit ribosomal RNA gene, partial sequence; internal transcribed spacer 1, 5.8S...	Aspergillus nidul...	1003	1003	98%	0.0	100.00%	553	MG459155.1
Aspergillus nidulans strain CMXY2059 small subunit ribosomal RNA gene, partial sequence; internal transcri...	Aspergillus nidul...	1003	1003	100%	0.0	99.46%	569	MG991576.1
Aspergillus nidulans strain JT4 small subunit ribosomal RNA gene, partial sequence; internal transcribed spa...	Aspergillus nidul...	1003	1003	100%	0.0	99.46%	589	MG734752.1
Aspergillus nidulans strain FGSC A4 small subunit ribosomal RNA gene, partial sequence; internal transcribe...	Aspergillus nidul...	1003	1003	100%	0.0	99.46%	1783	KY074657.1
Aspergillus sp. strain TJ29 small subunit ribosomal RNA gene, partial sequence; internal transcribed spacer ...	Aspergillus sp.	1003	1003	100%	0.0	99.46%	[illegible]	[illegible]
Aspergillus nidulans isolate HUIB03 small subunit ribosomal RNA gene, partial sequence; internal transcribe...	Aspergillus nidul...	1003	1003	100%	0.0	99.46%	[illegible]	[illegible]

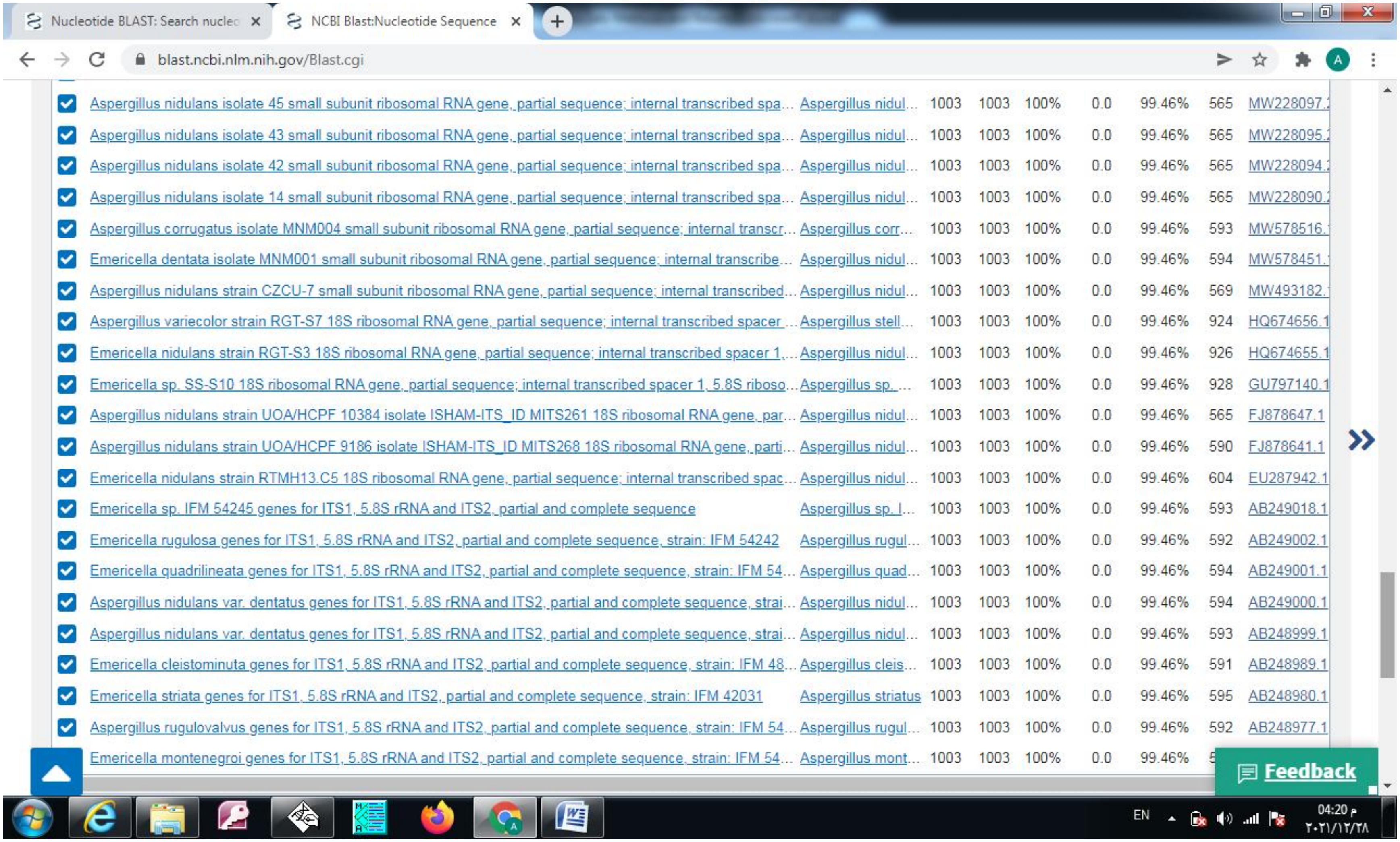

Aspergillus nidulans isolate 45 small subunit ribosomal RNA gene, partial sequence; internal transcribed spa...	Aspergillus nidul...	1003	1003	100%	0.0	99.46%	565	MW228097.2
Aspergillus nidulans isolate 43 small subunit ribosomal RNA gene, partial sequence; internal transcribed spa...	Aspergillus nidul...	1003	1003	100%	0.0	99.46%	565	MW228095.2
Aspergillus nidulans isolate 42 small subunit ribosomal RNA gene, partial sequence; internal transcribed spa...	Aspergillus nidul...	1003	1003	100%	0.0	99.46%	565	MW228094.2
Aspergillus nidulans isolate 14 small subunit ribosomal RNA gene, partial sequence; internal transcribed spa...	Aspergillus nidul...	1003	1003	100%	0.0	99.46%	565	MW228090.2
Aspergillus corrugatus isolate MNM004 small subunit ribosomal RNA gene, partial sequence; internal transcr...	Aspergillus corr...	1003	1003	100%	0.0	99.46%	593	MW578516.1
Emericella dentata isolate MNM001 small subunit ribosomal RNA gene, partial sequence; internal transcribe...	Aspergillus nidul...	1003	1003	100%	0.0	99.46%	594	MW578451.1
Aspergillus nidulans strain CZCU-7 small subunit ribosomal RNA gene, partial sequence; internal transcribed...	Aspergillus nidul...	1003	1003	100%	0.0	99.46%	569	MW493182.1
Aspergillus variecolor strain RGT-S7 18S ribosomal RNA gene, partial sequence; internal transcribed spacer ...	Aspergillus stell...	1003	1003	100%	0.0	99.46%	924	HQ674656.1
Emericella nidulans strain RGT-S3 18S ribosomal RNA gene, partial sequence; internal transcribed spacer 1,...	Aspergillus nidul...	1003	1003	100%	0.0	99.46%	926	HQ674655.1
Emericella sp. SS-S10 18S ribosomal RNA gene, partial sequence; internal transcribed spacer 1, 5.8S riboso...	Aspergillus sp. ...	1003	1003	100%	0.0	99.46%	928	GU797140.1
Aspergillus nidulans strain UOA/HCPF 10384 isolate ISHAM-ITS_ID MITS261 18S ribosomal RNA gene, par...	Aspergillus nidul...	1003	1003	100%	0.0	99.46%	565	FJ878647.1
Aspergillus nidulans strain UOA/HCPF 9186 isolate ISHAM-ITS_ID MITS268 18S ribosomal RNA gene, parti...	Aspergillus nidul...	1003	1003	100%	0.0	99.46%	590	FJ878641.1
Emericella nidulans strain RTMH13.C5 18S ribosomal RNA gene, partial sequence; internal transcribed spac...	Aspergillus nidul...	1003	1003	100%	0.0	99.46%	604	EU287942.1
Emericella sp. IFM 54245 genes for ITS1, 5.8S rRNA and ITS2, partial and complete sequence	Aspergillus sp. I...	1003	1003	100%	0.0	99.46%	593	AB249018.1
Emericella rugulosa genes for ITS1, 5.8S rRNA and ITS2, partial and complete sequence, strain: IFM 54242	Aspergillus rugul...	1003	1003	100%	0.0	99.46%	592	AB249002.1
Emericella quadrilineata genes for ITS1, 5.8S rRNA and ITS2, partial and complete sequence, strain: IFM 54...	Aspergillus quad...	1003	1003	100%	0.0	99.46%	594	AB249001.1
Aspergillus nidulans var. dentatus genes for ITS1, 5.8S rRNA and ITS2, partial and complete sequence, strai...	Aspergillus nidul...	1003	1003	100%	0.0	99.46%	594	AB249000.1
Aspergillus nidulans var. dentatus genes for ITS1, 5.8S rRNA and ITS2, partial and complete sequence, strai...	Aspergillus nidul...	1003	1003	100%	0.0	99.46%	593	AB248999.1
Emericella cleistominuta genes for ITS1, 5.8S rRNA and ITS2, partial and complete sequence, strain: IFM 48...	Aspergillus cleis...	1003	1003	100%	0.0	99.46%	591	AB248989.1
Emericella striata genes for ITS1, 5.8S rRNA and ITS2, partial and complete sequence, strain: IFM 42031	Aspergillus striatus	1003	1003	100%	0.0	99.46%	595	AB248980.1
Aspergillus rugulovalvus genes for ITS1, 5.8S rRNA and ITS2, partial and complete sequence, strain: IFM 54...	Aspergillus rugul...	1003	1003	100%	0.0	99.46%	592	AB248977.1
Emericella montenegroi genes for ITS1, 5.8S rRNA and ITS2, partial and complete sequence, strain: IFM 54...	Aspergillus mont...	1003	1003	100%	0.0	99.46%	[illegible]	[illegible]

Nucleotide BLAST: Search nucleo | NCBI Blast:Nucleotide Sequence | NCBI Blast:Nucleotide Sequence

blast.ncbi.nlm.nih.gov/Blast.cgi

Sequences producing significant alignments

Download | New Select columns | Show 100

select all *100 sequences selected*

GenBank | Graphics | Distance tree of results | New MSA Viewer

	Description	Scientific Name	Max Score	Total Score	Query Cover	E value	Per. Ident	Acc. Len	Accession
☑	Aspergillus quadrilineatus strain ATCC 16816 18S ribosomal RNA gene, partial sequence; internal transcribe...	Aspergillus qua...	1009	1009	100%	0.0	99.64%	590	AY373889.1
☑	Emericella quadrilineata strain ATCC 16816 18S ribosomal RNA gene, partial sequence; internal transcribed...	Aspergillus qua...	1009	1009	100%	0.0	99.64%	566	AY213642.1
☑	Aspergillus spinulosporus strain CBS 120.55 small subunit ribosomal RNA gene, partial sequence; internal tr...	Aspergillus spin...	1003	1003	100%	0.0	99.46%	590	MH857403.1
☑	Aspergillus corrugatus strain CBS 191.77 small subunit ribosomal RNA gene, partial sequence; internal tran...	Aspergillus corr...	998	998	100%	0.0	99.28%	1229	MH861048.1
☑	Aspergillus foveolatus CBS 279.81 ITS region; from TYPE material	Aspergillus fove...	998	998	100%	0.0	99.28%	614	NR_151791.1
☑	Aspergillus foveolatus culture-collection CBS:279.81 18S ribosomal RNA gene, partial sequence; internal tra...	Aspergillus fove...	998	998	100%	0.0	99.28%	732	KX423658.1
☑	Aspergillus fruticulosus strain CBS 486.65 small subunit ribosomal RNA gene, partial sequence; internal tran...	Aspergillus frutic...	994	994	100%	0.0	99.10%	885	MH858680.1
☑	Aspergillus corrugatus CBS 191.77 ITS region; from TYPE material	Aspergillus corr...	994	994	98%	0.0	99.45%	556	NR_151789.1
☑	Aspergillus quadrilineatus NRRL 201 ITS region; from TYPE material	Aspergillus qua...	994	994	97%	0.0	100.00%	587	NR_131289.1
☑	Aspergillus quadrilineatus isolate NRRL 201 18S ribosomal RNA gene, partial sequence; internal transcribed...	Aspergillus qua...	994	994	97%	0.0	100.00%	1124	EF652433.1
☑	Aspergillus rugulosus NRRL 206 ITS region; from TYPE material	Aspergillus rugu...	989	989	97%	0.0	99.81%	587	NR_131290.1
☑	Aspergillus rugulosus isolate NRRL 206 18S ribosomal RNA gene, partial sequence; internal transcribed spa...	Aspergillus rugu...	989	989	97%	0.0	99.81%	1124	EF652434.1
☑	Aspergillus microcysticus strain CBS 120.58 small subunit ribosomal RNA gene, partial sequence; internal tr...	Aspergillus micr...	987	987	99%	0.0	98.92%	567	MH857716.1
☑	Emericella nidulans 18S ribosomal RNA gene, partial sequence; internal transcribed spacer 1, 5.8S ribosom...	Aspergillus nidul...	987	987	100%	0.0	98.92%	565	AF138289.1
☑	Aspergillus falconensis CBS 271.91 ITS region; from TYPE material	Aspergillus falco...	985	985	98%	0.0	99.09%	558	NR_151790.1
☑	Aspergillus latus CBS 492.65 ITS region; from TYPE material	Aspergillus latus	985	985	100%	0.0	98.74%	565	NR_154682.1
☑	Aspergillus nidulans ATCC 10074 ITS region; from TYPE material	Aspergillus nidul...	985	985	98%	0.0	99.45%	[illegible]	[illegible]
	Aspergillus striatus NRRL 4699 ITS region; from TYPE material	Aspergillus striat...	983	983	97%	0.0	99.63%	[illegible]	[illegible]

Feedback

EN 04:21 م ٢٠٢١/١٢/٢٨
