# Supplementary material for: The removal of black ink via Emericella quadrilineata as a green alternative technique to recycling ink waste papers
Source: PLoS One. 2025 May 29;20(5):e0324022. doi: 10.1371/journal.pone.0324022 (PMC12122025; doi:10.1371/journal.pone.0324022)
Supplement: S1 Table — (PDF) [file pone.0324022.s002.pdf]

**Fungal isolates**	**Deinking ability (%)**	
	**Means**	**± SD**
Aspergillus sp	**90**	**±2**
Cladosporium sp	**86**	**± 3**
Fusarium sp	**82**	**± 1**
Penicillium sp	**75**	**± 1**
Rhizopus sp	**72**	**± 0.5**
