# Supplementary material for: The removal of black ink via Emericella quadrilineata as a green alternative technique to recycling ink waste papers
Source: PLoS One. 2025 May 29;20(5):e0324022. doi: 10.1371/journal.pone.0324022 (PMC12122025; doi:10.1371/journal.pone.0324022)
Supplement: S2 Table — (PDF) [file pone.0324022.s003.pdf]

| Temperature(℃) | Deinking ability (%) ±SD | | |
|---|---|---|---|
| | 3 days | 6 days | 9 days |
| 20 | 75 ± 0.5 | 80 ± 1 | 85 ± 0.9 |
| 25 | 80 ± 1 | 85 ± 2 | 90 ± 1 |
| 30 | 90 ± 3 | 92 ± 2 | 94 ± 1 |
| 35 | 60 ± 0.5 | 65 ± 0.9 | 70 ± 1.5 |
| 40 | 45 ± 2 | 50 ± 0.95 | 55 ± 1 |

| pH | Deinking ability (%) ±SD | | |
|---|---|---|---|
| | 3 days | 6 days | 9 days |
| 2 | 65 ±2 | 70 ± 0.95 | 75 ± 1 |
| 4 | 75 ± 0.5 | 80 ± 0.9 | 85 ± 1.5 |
| 6 | 85 ±3 | 90 ± 2 | 93 ± 1 |
| 7 | 80 ±1 | 85 ± 2 | 92 ± 1 |
| 8 | 65 ± 0.5 | 70 ± 1 | 75 ± 0.9 |
| 10 | 56 ± 3 | 60 ± 2 | 65 ±0.95 |

| **Ink Concentrations (mg/L)** | **Deinking ability (%) ±SD** | | |
|---|---|---|---|
| | **3 days** | **6 days** | **9 days** |
| **5000** | **94 ± 0.5** | **95 ± 0.9** | **95 ± 0.5** |
| **10000** | **95 ± 0.55** | **96 ± 0.95** | **96 ± 0.5** |
| **20000** | **96 ± 0.8** | **97 ± 0.5** | **97 ± 0.7** |
| **30000** | **92 ± 1.5** | **93 ± 1** | **93 ± 0.9** |
| **40000** | **91 ± 2** | **93 ± 0.6** | **93 ± 0.9** |
| **50000** | **91 ± 1** | **92 ± 2** | **92 ± 1.5** |

| **Inoculum dose (discs)** | **Deinking ability (%) ±SD** | | |
|---|---|---|---|
| | **3 days** | **6 days** | **9 days** |
| **One** | **94 ± 0.9** | **95 ± 0.5** | **95 ± 1** |
| **Two** | **95 ± 1.5** | **96 ± 2** | **96 ± 1** |
| **Three** | **96 ± 2** | **97 ± 2.1** | **97 ± 1** |
